# Bionic Aerogel with a Lotus Leaf-like Structure for Efficient Oil-Water Separation and Electromagnetic Interference Shielding

**DOI:** 10.3390/gels9030214

**Published:** 2023-03-10

**Authors:** Fengqi Liu, Yonggang Jiang, Junzong Feng, Liangjun Li, Jian Feng

**Affiliations:** Science and Technology on Advanced Ceramic Fibers and Composites Laboratory, College of Aerospace Science and Technology, National University of Defense Technology, Changsha 410073, China

**Keywords:** bionic aerogel, oil-water separation, electromagnetic interference shielding, lotus leaf-like structure

## Abstract

Increasing pollution from industrial wastewater containing oils or organic solvents poses a serious threat to both the environment and human health. Compared to complex chemical modifications, bionic aerogels with intrinsic hydrophobic properties exhibit better durability and are considered as ideal adsorbents for oil-water separation. However, the construction of biomimetic three-dimensional (3D) structures by simple methods is still a great challenge. Here, we prepared biomimetic superhydrophobic aerogels with lotus leaf-like structures by growing carbon coatings on Al_2_O_3_ nanorod-carbon nanotube hybrid backbones. Thanks to its multicomponent synergy and unique structure, this fascinating aerogel can be directly obtained through a simple conventional sol-gel and carbonization process. The aerogels exhibit excellent oil-water separation (22 g·g^−1^), recyclability (over 10 cycles) and dye adsorption properties (186.2 mg·g^−1^ for methylene blue). In addition, benefiting from the conductive porous structure, the aerogels also demonstrate outstanding electromagnetic interference (EMI) shielding capabilities (~40 dB in X-band). This work presents fresh insights for the preparation of multifunctional biomimetic aerogels.

## 1. Introduction

In recent years, oil and organic pollutant discharges from growing industries such as oil extraction, metal processing, textiles and food manufacturing not only cause serious damage to water resources, but also pose a threat to the survival of marine life and human health [1]. Traditional wastewater treatment technologies based on physics (adsorption, membrane separation), chemistry (combustion, redox) and biology (biodegradation) have gradually struggled to meet the increasing demand due to low efficiency, high cost and susceptibility to secondary pollution [2,3]. Therefore, the search for efficient oil pollution treatment technologies and adsorbents has become the focus of researchers.

Aerogels possess low density, high specific surface area, high porosity and adjustable hydrophobicity, which can serve as a promising material for adsorption [4]. Currently, a variety of aerogel materials have been developed for oil-water separation applications, mainly including biomass-based aerogels [5], carbon-based aerogels [6], polymer aerogels [7] and metal-organic frameworks [8]. However, due to limited hydrophobicity and adsorption properties, aerogels usually suffer from complex chemical modifications to achieve the desired application. Grafting chemical groups such as siloxanes and fluorosilanes on the surface of aerogels is the main way of chemical modification [9,10,11], which not only involves time-consuming processes (chemical vapor deposition, impregnation, etc.) but also results in hydrophobicity with poor stability in strong corrosive environments [12].

Inspired by natural organisms, such as lotus leaves, duck feathers and shark skin, researchers have successfully fabricated a variety of biomimetic superhydrophobic materials by morphology and property mimicry [1,13]. Low surface energy composition and high roughness structure are two important features for the construction of bionic hydrophobic surfaces [14]. Kevin et al. [15] found that poly(dimethylsiloxane) (PDMS) hydrophobic coatings prepared on low surface energy aluminum substrates have better de-icing properties compared to other substrates, which triggered extensive interest in the construction of Al_2_O_3_-based hydrophobic structures. Karthik et al. [16] prepared superhydrophobic composite coatings (hydrophobic angle 154°) on Al_2_O_3_ surfaces by a combination of electroless copper deposition and lauramine surface modification. Luo et al. [17] reported a solid-state spraying method for constructing discontinuous reef-like hydrophobic layers (152°) on Al_2_O_3_ surfaces, exhibiting excellent frictional durability. Fu et al. [18] successively obtained structures with better hydrophobic properties by ionomer electrolytic oxidation (PEO), chemical vapor deposition and chemical modification with fluorosilanes (160.5). Further, thanks to a rational structural design, Yang et al. [19] fabricated T-shaped micro/nanostructured Al_2_O_3_ hydrophobic coatings directly by the PEO method.

In addition, physical methods such as vacuum deposition [20], laser ablation [21], sputtering [22], atomic layer deposition [23,24] and chemical methods such as chemical vapor deposition [25] and spray pyrolysis [26] are also widely used to prepare a variety of biomimetic hydrophobic surfaces, but these techniques have strict requirements on equipment and are difficult to produce on a large scale. Moreover, these methods currently focus on building superhydrophobic surfaces, which are difficult to combine with the sol-gel technology to prepare superhydrophobic biomimetic three-dimensional (3D) aerogel materials.

On the other hand, in addition to water pollution, the electromagnetic interference (EMI) pollution caused by electronic and 5G communication devices has become unignorable in the information age [27]. EMI shielding materials with light weight, robustness and high absorption rate has attracted considerable attention [28,29]. The EMI effectiveness (SE) is the ability of a material to attenuate electromagnetic waves passing through it and is highly positively correlated with the electrical conductivity of materials [30]. In our previous work, core-shell nanorod aerogels with intrinsic superhydrophobicity were prepared [31]. However, the brittleness and unsatisfactory electrical conductivity of the core-shell aerogel are still the main obstacles for its use in oil-water separation and EMI shielding.

Here, to overcome the above obstacles, biomimetic superhydrophobic aerogels with lotus-like structures were prepared by wrapping rough carbon layers on Al_2_O_3_ nanorod-carbon nanotube hybrid skeletons. Thanks to the enhanced mechanical properties and electrical conductivity brought by carbon nanotubes (CNTs), the aerogels exhibit excellent oil-water separation, acid/alkali resistance, recyclability, dye adsorption and EMI shielding properties. In this work, bionic structured aerogels are developed by a simple process without post-chemical modification, providing a new perspective for the fabrication of multifunctional aerogels for efficient oil-water separation and EMI shielding.

## 2. Results and Discussion

### 2.1. Characterization of Carbon Layer Wrapped Al_2_O_3_ Nanorods-Carbon Nanotubes Hybrid Aerogels (CACAs)

**Figure 1:**
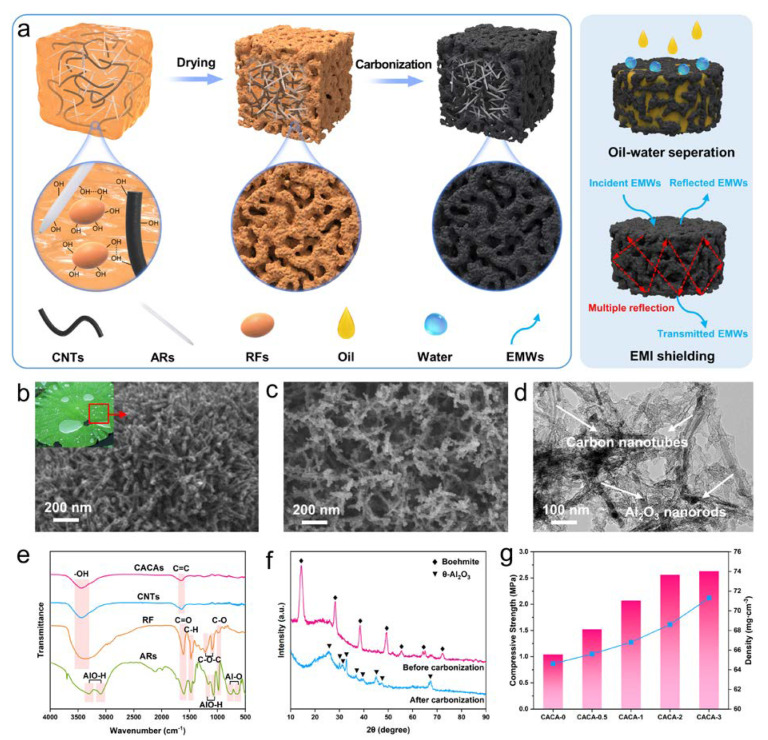
(**a**) Schematic diagram of CACAs preparation process. (**b**) Macro photo (inset) and SEM image of lotus leaves [32]. Copyright 2019, Elsevier. (**c**) SEM and (**d**) TEM images of CACAs. (**e**) FT-IR patterns of raw materials and CACAs. (**f**) XRD patterns before and after carbonization. (**g**) Compression strength of CACAs with different CNTs content.

As shown in Figure 1a, biomimetic CACAs can be obtained after gelation, drying and high temperature carbonization of the mixture of Al_2_O_3_ nanorod sols, RF sols and CNTs. The micron-scale Al_2_O_3_ nanorod-carbon nanotube hybrid skeleton with nano-rough carbon layer constitutes a rod-like micro-nano structure similar to the surface of the lotus leaves (Figure 1b,c) [32]. The TEM image also shows that the Al_2_O_3_ nanorods (Ars) and CNTs are entangled with each other and uniformly covered by the carbon layer (Figure 1d). As can be seen from the FT-IR patterns (Figure 1e), the Ars exhibit typical Al_2_O_3_ absorption peaks [33], including the stretching vibration peaks of AlO-H (3099 and 3285 cm^−1^), the bending vibration peaks of AlO-H (1070 and 1162 cm^−1^) and the torsional vibration peaks of Al-O (650 and 760 cm^−1^). The absorption peaks at 1611, 1478 and 981 cm^−1^ are attributed to C=O, C-H and C-O, respectively, originating from organic solvents in RF sols and Al_2_O_3_ nanorod sols [31]. In addition, benefiting from the abundant -OH (peaks at 3400 cm^−1^) on the surface of RF molecules and CNTs, intermolecular interactions with Ars can be established, allowing the carbon layer to grow uniformly on the hybridized backbone (Figure 1a). After carbonization, only C=C peak from the benzene ring and -OH peak from moisture in air are observed due to the strong light absorption effect of carbon. After the high temperature carbonization process, the Ars transform from boehmite phase to θ-Al_2_O_3_ (Figure 1f), while the enhancement of the broad peak at about 25° represents the transformation of RF to carbon layers. As seen in Figure 1g, with the increase in CNTs content, the density of CACAs increased monotonically from 64.6 to 71.3 mg·cm^−3^. However, the compressive strength first increased rapidly from 1.04 Mpa for CACA-0 to 2.56 Mpa for CACA-2, and then elevated slowly to 2.63 Mpa for CACA-3, which was attributed to the inhomogeneous dispersion of CNTs at high concentration (Figure 1g and Figure S1). Notably, the fracture strain of CACAs also increased significantly from 13.8% for CACA-0 to 27.3% for CACA-2, with the increase in CNTs content indicating a significant improvement of toughness. Therefore, all oil-water separation performance experiments were conducted based on CACA-2.

### 2.2. Oil-Water Separation Properties of CACAs

**Figure 2:**
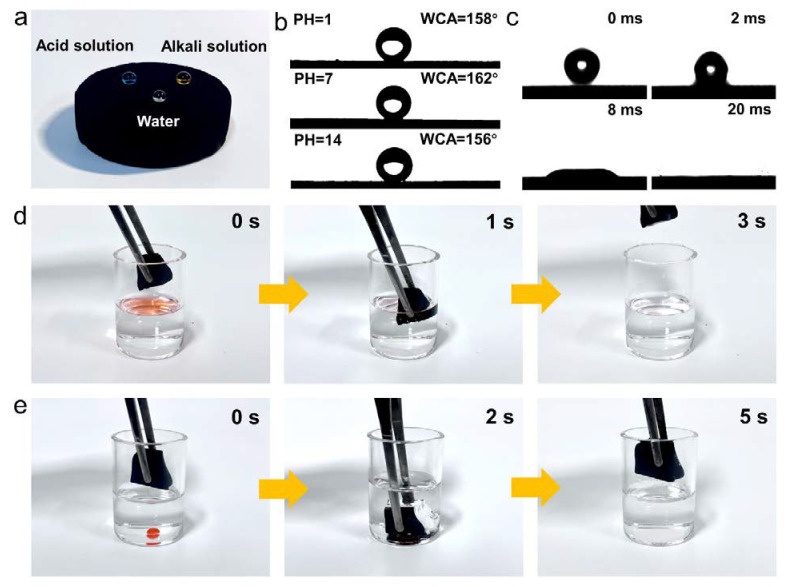
(**a**) Superhydrophobicity and (**b**) WCA of CACAs towards liquids with different PH. (**c**) Dynamic adsorption process of chloroform by CACAs. The adsorption processes of (**d**) n-hexane and (**e**) chloroform (both stained with Sudan red) from the surface and bottom of the water, respectively.

As shown in Figure 2a,b, hydrochloric acid droplets (PH = 1, stained by methylene blue), sodium hydroxide droplets (PH = 14, stained by methyl orange) and transparent water droplets (PH = 7) can stand stably on the sample surface in a nearly spherical shape with water contact angles (WCA) of 158°, 156° and 162°, respectively, indicating the excellent superhydrophobicity and chemical durability of the CACAs. The high temperature treated carbon layer not only possesses low surface energy and nano-roughness, but also forms a lotus-like micro-nano structure together with the Al_2_O_3_ nanorod-carbon nanotube backbone. The rough surface of CACAs is in point contact with water droplets and forms a Cassie-Baxter model, which means that a large number of air pockets are trapped at the interface to avoid wetting of water droplets, thus forming a superhydrophobic surface [34,35]. In addition, compared to unstable chemical modifications, the carbon layer with excellent chemical stability can effectively prevent the internal material from being corroded by acids or alkalis [36], laying the foundation for applications in harsh environments. The whole process of oil absorption by CACAs is monitored by a high-speed camera and found to take only 35 ms from droplet contact with the material to complete absorption, demonstrating ultra-fast oil absorption rate and super lipophilicity (Figure 2c). As shown in Figure 2d,e, both light oil (n-hexane) floating on the surface and heavy olive oil sunk underwater can be absorbed by CACAs within a few seconds. Owning to the superhydrophobicity, the aerogel that comes up from the bottom of the water is not wetted by water, which undoubtedly offers a facile and energy-saving absorption method for practical wastewater treatment.

**Figure 3:**
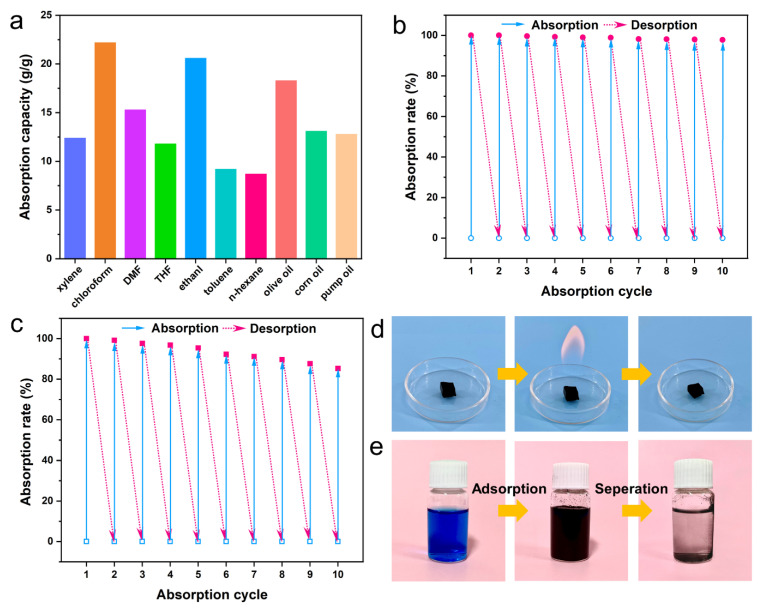
(**a**) The absorption capacity of CACAs towards different types of oils and organic solvents. Absorption recyclability of the CACAs through (**b**) distillation (n-hexane) and (**c**) combustion (DMF) for desorption. (**d**) Combustion cycle process of CACAs for DMF. (**e**) Adsorption and separation process of CACAs for methylene blue.

Figure 3a shows the adsorption capacity of CACA-2 for various kinds of organic solvents (e.g., xylene, toluene, n-hexane, THF, DMF, and chloroform) and oils (e.g., pump oil, silicone oil, and olive oil), which are the common pollutants in water resources, with values in the range of 12 to 22 g·g^−1^ depending on the density and viscosity of the pollutants. Compared to previously reported porous foams or aerogels (Table 1) [10,11,37,38,39], CACA exhibits similar adsorption capacity and better hydrophobicity, but it is prepared by a simpler method. The durability of the adsorbent and the collectability of the pollutants are also essential indicators for adsorbent material. Taking low boiling n-hexane as an example, it can be easily removed from the adsorbent material and re-collected by distillation at 80 °C. After 10 cycles, CACAs exhibit excellent cycling stability and the adsorption capacity only slightly decreased by 2% (Figure 3b), which is attributed to the robust hybrid aerogel backbone that can resist the surface tension of solvent volatilization [40]. For high boiling point sorbents, CACAs can be ignited in air after adsorption to remove the adsorbate by combustion (Figure 3d). Benefiting from the excellent flame retardancy and thermal stability, the absorption rate of CACAs for DMF remained at 85% after 10 adsorption-combustion cycles, indicating its excellent reusability (Figure 3c). Moreover, CACAs with high specific surface area (SSA) also exhibited excellent adsorption performance for dyes such as methyl orange and methylene blue. After the addition of 10 mg CACAs powder for 15 min, the methylene blue solution (10 mg·L^−1^) changed from blue to clear after centrifugation (Figure 3e). By putting the recovered CACAs into ethanol, the adsorbed methylene blue is released again, facilitating the subsequent green recycling of the contaminant. According to the Langmuir isothermal adsorption curve (Figures S2 and S3) [41,42], the adsorption capacity of CACAs for methylene blue was as high as 186.2 mg·g^−1^ (Detailed discussion can be seen in Supplementary Materials).

### 2.3. EMI Shielding Properties

Al_2_O_3_ is known to be non-conductive and transparent to electromagnetic waves [43], but this situation changes for CACAs because the conducting carbon nanotubes form an interpenetrating network with Al_2_O_3_ nanorods, coupled with a tightly clinging carbon layer, constituting a 3D conducting network for transitions and hopping of electrons [44]. According to electromagnetic theory, the total shielding effectiveness (SE_T_) usually governed by absorption (SE_A_), reflection (SE_R_) and transmission through multiple internal reflections (SE_M_), where SE_M_ can be neglected in this paper because SE_A_ is greater than −10 dB [30]. For non-magnetic materials, a higher conductivity means a more severe impedance mismatch between the material and air, resulting in increased reflections at the corresponding interfaces and thus enhancing the total EMI shielding effect [44,45].

**Figure 4:**
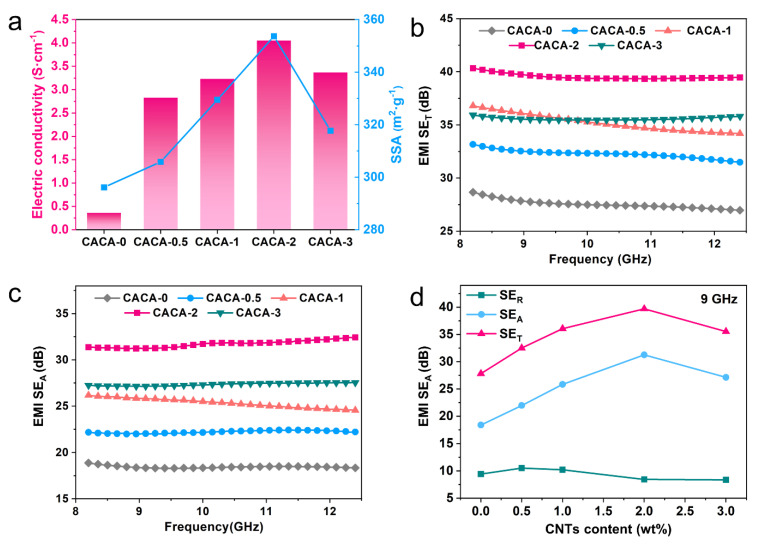
(**a**) Conductivity and SSA values of CACAs with different CNTs contents. (**b**) EMI SE_T_ and (**c**) SE_A_ properties of CANAs with various CNTs contents measured in X-band. (**d**) EMI SE_T,_ SE_R_ and SE_A_ value of CANAs with various CNTs contents at 9 GHz.

Figure 4a illustrates the conductivity of CACAs with different carbon nanotube contents, and it can be found that CACAs-2 has the highest conductivity of 4.05 S·cm^−1^; thus, it is expected to be a promising EMI shielding material. The EMI SE_T_ and SE_A_ of CACAs with different CNTs loadings were measured over the frequency of 8.2–12.4 GHz (X band) (Figure 4b,c). The CANAs-0 obtains an SE_T_ of ~28 dB with SE_R_ values of ~18 dB, which is higher than the basic requirement for commercial EM shielding materials (20 dB). After adding 2% CNTs, the value of SE_T_ reaches to 40.2 dB with higher SE_A_ (~32.4 dB). However, for sample CACA-3, the agglomeration of CNTs caused the degradation of the electrical and EMI SE properties of the hybrid aerogel (SE_T_ ≈ 35.5 dB), which can be confirmed in the decrease in conductivity and SSA (Figure 4a). Figure 4d presents the SE_T_, SE_A_ and SE_R_ of CANAs with various CNTs loadings at a frequency of 9 GHz. It can be seen that both SE_T_ and SE_A_ show an upward trend with the increase in CNTs loading (below 2%), while SE_R_ remains almost constant. In addition, SE_A_ contributes much more to SE_T_ than SE_R_, indicating that absorption is the main shielding mechanism.

As we know, EMI shielding mechanism of metals and other highly conductive materials are mainly reflection rather than absorption [46]. In contrast, CANAs in this work are dominated by absorption mechanism, which is attributed to the high SSAs and high porosity that can trap more EM radiation inside the material [47]. The repeated reflection of EM waves (EMWs) inside the material pores leads to the dissipation of their energy in the form of heat, thus increasing the SE_A_ (Figure 1a) [44]. With the increased loadings of highly conductive CNTs, more interfaces and heterogeneous systems are also formed in the material, which enhance the scattering of EMWs and contribute more to the absorption enhancement.

## 3. Conclusions

In summary, the carbon layer with nano-roughness and the Al_2_O_3_ nanorod-carbon nanotube skeleton constitute a lotus-like hydrophobic structure. With the addition of carbon nanotubes, the biomimetic aerogels exhibit enhanced mechanical properties (2.56 MPa) and electrical conductivity (4.05 S·cm^−1^), permitting them to demonstrate excellent comprehensive properties, including oil-water separation (22 g·g^−1^), corrosion resistance (WCA > 156° in the pH range of 1–14), recyclability (over 10 cycles), dye adsorption (186.2 mg·g^−1^ for methylene blue) and EMI shielding (~40 dB in X-band) properties. The multifunctional CACAs not only provide a reference for the simple preparation of bionic materials, but also show great potential to deal with water pollution and electromagnetic pollution.

## 4. Materials and Methods

Al_2_O_3_ nanorod sols and resorcinol-formaldehyde (RF) sols were synthesized in the same way as previous work. An amount of 10 g Al_2_O_3_ nanorod sols, 10 g deionized water, 4 g RF sols and different masses of CNTs were mixed thoroughly and then gelled and aged at 80 °C for 48 h. The wet gel was placed in ethanol for solvent displacement for 72 h, and the solvent was refreshed every 24 h. Finally, the samples were subjected to CO_2_ supercritical drying and high temperature carbonization (treated at 1400 °C in argon atmosphere for 2 h with a heating rate of 5 °C·min^−1^) to obtain carbon layer wrapped Al_2_O_3_ nanorods-carbon nanotubes hybrid aerogels (CACAs). The CNTs content was defined as the mass ratio of CNTs to ARs. In the end, 0.5%, 1%, 2% and 3% of CNTs content were named as CACA-0.5, CACA-1, CACA-2 and CACA-3, respectively. Samples without carbon nanotubes were defined as CACA-0. Further experimental details including characterization, measurement of methylene blue standard working curve and Langmuir adsorption isotherm curves can be found in the Supplementary Materials.

## Figures and Tables

**Table 1:** Comparison of different superhydrophobic porous materials for oil adsorption.

Materials	WCA (°)	Adsorption Capacity (g·g^−1^)	Hydrophobic Agent	Post-Treatment Methods	Ref.
Poly (lactic acid) foams	141	8.1–13.3	Fluorosiloxane	CVD	[10]
Activated carbon aerogel	137.6–145.6	4.06–12.31	Polydimethylsiloxane (PDMS)	Impregnation	[11]
Fe_3_O_4_@PANI/chitosan composite aerogel	121	2–21	Methyltrichlorosilane (MTCS)	CVD	[37]
HLNPs/Fe@C@Ti@PS sponge	149.7–153.3	17.17–24.78	Polydimethylsiloxane (PDMS)	Spraying and impregnation	[38]
Polyurethane-Cu sponge	171	13–18	AgNO_3_ and n-dodecanoic acid	Impregnation	[39]
CACAs	156–162	12–22	/	/	This work

## Data Availability

The data presented in this study are available on request from the corresponding author.

## Supplementary Materials

The following supporting information can be downloaded at: https://www.mdpi.com/article/10.3390/gels9030214/s1, Figure S1: the compressive stress-strain curves of CACAs with different CNTs contents; Figure S2: (a) standard working curve of methylene blue; (b) fitting curve of Langmuir adsorption isotherm equation.

## References

[1] Hou L., Wang N., Wu J., Cui Z., Jiang L., Zhao Y. (2018). Bioinspired Superwettability Electrospun Micro/Nanofibers and Their Applications. Adv. Funct. Mater..

[2] Ganesamoorthy R., Vadivel V.K., Kumar R., Kushwaha O.S., Mamane H. (2021). Aerogels for Water Treatment: A Review. J. Cleaner Prod..

[3] Mariana M., Abdul Khalil H.P.S., Yahya E.B., Olaiya N.G., Alfatah T., Suriani A.B., Mohamed A. (2022). Recent Trends and Future Prospects of Nanostructured Aerogels in Water Treatment Applications. J. Water Process Eng..

[4] Zhu L., Zong L., Wu X., Li M., Wang H., You J., Li C. (2018). Shapeable Fibrous Aerogels of Metal–Organic-Frameworks Templated with Nanocellulose for Rapid and Large-Capacity Adsorption. ACS Nano.

[5] Qin H., Zhang Y., Jiang J., Wang L., Song M., Bi R., Zhu P., Jiang F. (2021). Multifunctional Superelastic Cellulose Nanofibrils Aerogel by Dual Ice-Templating Assembly. Adv Funct. Mater..

[6] Lee J.-H., Park S.-J. (2020). Recent Advances in Preparations and Applications of Carbon Aerogels: A Review. Carbon.

[7] Wang N.-N., Wang H., Wang Y.-Y., Wei Y.-H., Si J.-Y., Yuen A.C.Y., Xie J.-S., Yu B., Zhu S.-E., Lu H.-D. (2019). Robust, Lightweight, Hydrophobic, and Fire-Retarded Polyimide/MXene Aerogels for Effective Oil/Water Separation. ACS Appl. Mater. Interfaces.

[8] Gu J., Fan H., Li C., Caro J., Meng H. (2019). Robust Superhydrophobic/Superoleophilic Wrinkled Microspherical MOF@rGO Composites for Efficient Oil–Water Separation. Angew. Chem..

[9] Zu G., Kanamori K., Nakanishi K., Lu X., Yu K., Huang J., Sugimura H. (2019). Superelastic Multifunctional Aminosilane-Crosslinked Graphene Aerogels for High Thermal Insulation, Three-Component Separation, and Strain/Pressure-Sensing Arrays. ACS Appl. Mater. Interfaces.

[10] Ding H., Yang W., Yu W., Liu T., Wang H., Xu P., Lin L., Ma P. (2021). High Hydrophobic Poly(Lactic Acid) Foams Impregnating One-Step Si–F Modified Lignin Nanoparticles for Oil/Organic Solvents Absorption. Compos. Commun..

[11] Huang Y., Wu Y., Tao H., Yuan B. (2022). Bio-Based Porous Aerogel with Bionic Structure and Hydrophobic Polymer Coating for Efficient Absorption of Oil/Organic Liquids. Polymers.

[12] Zhou H., Wang H., Niu H., Gestos A., Wang X., Lin T. (2012). Fluoroalkyl Silane Modified Silicone Rubber/Nanoparticle Composite: A Super Durable, Robust Superhydrophobic Fabric Coating. Adv. Mater..

[13] Zhou H., Wang H., Niu H., Lin T. (2018). Recent Progress in Durable and Self-Healing Super-Nonwettable Fabrics. Adv. Mater..

[14] Domingues E.M., Arunachalam S., Nauruzbayeva J., Mishra H. (2018). Biomimetic Coating-Free Surfaces for Long-Term Entrapment of Air under Wetting Liquids. Nat. Commun..

[15] Golovin K., Dhyani A., Thouless M.D., Tuteja A. (2019). Low–Interfacial Toughness Materials for Effective Large-Scale Deicing. Science.

[16] Karthik N., Edison T.N.J.I., Lee Y.R., Sethuraman M.G. (2017). Fabrication of Corrosion Resistant Mussel-Yarn like Superhydrophobic Composite Coating on Aluminum Surface. J. Taiwan Inst. Chem. Eng..

[17] Luo X., Li C. (2019). Bioinspired Mechanically Robust Metal-Based Water Repellent Surface Enabled by Scalable Construction of a Flexible Coral-Reef-Like Architecture. Small.

[18] Fu J., Sun Y., Ji Y., Zhang J. (2022). Fabrication of Robust Ceramic Based Superhydrophobic Coating on Aluminum Substrate via Plasma Electrolytic Oxidation and Chemical Vapor Deposition Methods. J. Mater. Process. Technol..

[19] Yang C., Cui S., Weng Y., Wu Z., Liu L., Ma Z., Tian X., Fu R.K.Y., Chu P.K., Wu Z. (2021). Scalable Superhydrophobic T-Shape Micro/Nano Structured Inorganic Alumina Coatings. Chem. Eng. J..

[20] Ennaceri H., Wang L., Erfurt D., Riedel W., Mangalgiri G., Khaldoun A., El Kenz A., Benyoussef A., Ennaoui A. (2016). Water-Resistant Surfaces Using Zinc Oxide Structured Nanorod Arrays with Switchable Wetting Property. Surf. Coat. Technol..

[21] Pan S., Kota A.K., Mabry J.M., Tuteja A. (2013). Superomniphobic Surfaces for Effective Chemical Shielding. J. Am. Chem. Soc..

[22] Ou J., Fang G., Li W., Amirfazli A. (2019). Wetting Transition on Textured Surfaces: A Thermodynamic Approach. J. Phys. Chem. C.

[23] Korhonen J.T., Kettunen M., Ras R.H.A., Ikkala O. (2011). Hydrophobic Nanocellulose Aerogels as Floating, Sustainable, Reusable, and Recyclable Oil Absorbents. ACS Appl. Mater. Interfaces.

[24] Yuan D., Zhang T., Guo Q., Qiu F., Yang D., Ou Z. (2018). Recyclable Biomass Carbon@SiO_2_@MnO_2_ Aerogel with Hierarchical Structures for Fast and Selective Oil-Water Separation. Chem. Eng. J..

[25] Faustini M., Ceratti D.R., Louis B., Boudot M., Albouy P.-A., Boissière C., Grosso D. (2014). Engineering Functionality Gradients by Dip Coating Process in Acceleration Mode. ACS Appl. Mater. Interfaces.

[26] Tarwal N.L., Patil P.S. (2010). Superhydrophobic and Transparent ZnO Thin Films Synthesized by Spray Pyrolysis Technique. Appl. Surf. Sci..

[27] Agrawal P.R., Kumar R., Teotia S., Kumari S., Mondal D.P., Dhakate S.R. (2019). Lightweight, High Electrical and Thermal Conducting Carbon-RGO Composites Foam for Superior Electromagnetic Interference Shielding. Compos. Part B Eng..

[28] Xu J., Li R., Ji S., Zhao B., Cui T., Tan X., Gou G., Jian J., Xu H., Qiao Y. (2021). Multifunctional Graphene Microstructures Inspired by Honeycomb for Ultrahigh Performance Electromagnetic Interference Shielding and Wearable Applications. ACS Nano.

[29] Liang L., Li Q., Yan X., Feng Y., Wang Y., Zhang H.-B., Zhou X., Liu C., Shen C., Xie X. (2021). Multifunctional Magnetic Ti_3_C_2_T*_x_* MXene/Graphene Aerogel with Superior Electromagnetic Wave Absorption Performance. ACS Nano.

[30] Li Q., Chen L., Ding J., Zhang J., Li X., Zheng K., Zhang X., Tian X. (2016). Open-Cell Phenolic Carbon Foam and Electromagnetic Interference Shielding Properties. Carbon.

[31] Liu F., He C., Jiang Y., Yang Y., Peng F., Liu L., Men J., Feng J., Li L., Tang G. (2023). Carbon Layer Encapsulation Strategy for Designing Multifunctional Core-Shell Nanorod Aerogels as High-Temperature Thermal Superinsulators. Chem. Eng. J..

[32] Li X., Gong F., Liu D., He S., Yuan H., Dai L., Cai X., Liu J., Guo J., Jin Y. (2019). A Lotus Leaf Based Random Laser. Org. Electron..

[33] Liu F., Jiang Y., Peng F., Feng J., Li L., Feng J. (2023). Fiber-Reinforced Alumina-Carbon Core-Shell Aerogel Composite with Heat-Induced Gradient Structure for Thermal Protection up to 1800 °C. Chem. Eng. J..

[34] Tuteja A., Choi W., Ma M., Mabry J.M., Mazzella S.A., Rutledge G.C., McKinley G.H., Cohen R.E. (2007). Designing Superoleophobic Surfaces. Science.

[35] Fan Q., Lu T., Deng Y., Zhang Y., Ma W., Xiong R., Huang C. (2022). Bio-Based Materials with Special Wettability for Oil-Water Separation. Sep. Purif. Technol..

[36] Feng S., Luo W., Wang L., Zhang S., Guo N., Xu M., Zhao Z., Jia D., Wang X., Jia L. (2019). Preparation and Property of Extremely Stable Superhydrophobic Carbon Fibers with Core-Shell Structure. Carbon.

[37] Li S.-L., Wang Y.-T., Liu B.-W., Shi H.-G., Zhao H.-B., Wang Y.-Z. (2022). Fe3O4@PANI/Chitosan Composite Aerogel with Electromagnetic Induction Heating Capacity toward Efficient Removing Viscous Oil. Compos. Commun..

[38] Huang J., Li M., Ren C., Huang W., Miao Y., Wu Q., Wang S. (2023). Construction of HLNPs/Fe3O4 Based Superhydrophobic Coating with Excellent Abrasion Resistance, UV Resistance, Flame Retardation and Oil Absorbency. J. Environ. Chem. Eng..

[39] Zhu Q., Pan Q., Liu F. (2011). Facile Removal and Collection of Oils from Water Surfaces through Superhydrophobic and Superoleophilic Sponges. J. Phys. Chem. C.

[40] Zang X., Cao X., Zheng W., Zhu T., Lei Y., Huang J., Chen Z., Teng L., Bian J., Lai Y. (2023). Meniscus Inspired Flexible Superhydrophobic Coating with Remarkable Erosion Resistance for Pipeline Gas Transmission. Chem. Eng. J..

[41] Shao X., Lu W., Zhang R., Pan F. (2013). Enhanced Photocatalytic Activity of TiO_2_-C Hybrid Aerogels for Methylene Blue Degradation. Sci. Rep..

[42] Zhang P., An Q., Guo J., Wang C.-C. (2013). Synthesis of Mesoporous Magnetic Co-NPs/Carbon Nanocomposites and Their Adsorption Property for Methyl Orange from Aqueous Solution. J. Colloid Interface Sci..

[43] Shang X., Zhai D., Zhang F., Wei C., Chen J., Liu M., Peng J. (2019). Electromagnetic Waves Transmission Performance of Alumina Refractory Ceramics in 2.45 GHz Microwave Heating. Ceram. Int..

[44] Patle V.K., Kumar R., Sharma A., Dwivedi N., Muchhala D., Chaudhary A., Mehta Y., Mondal D.P., Srivastava A.K. (2020). Three Dimension Phenolic Resin Derived Carbon-CNTs Hybrid Foam for Fire Retardant and Effective Electromagnetic Interference Shielding. Compos. Part C Open Access..

[45] Kumar R., Jain H., Sriram S., Chaudhary A., Khare A., Ch V.A.N., Mondal D.P. (2020). Lightweight Open Cell Aluminum Foam for Superior Mechanical and Electromagnetic Interference Shielding Properties. Mater. Chem. Phys..

[46] Li X., Yin X., Liang S., Li M., Cheng L., Zhang L. (2019). 2D Carbide MXene Ti_2_CTX as a Novel High-Performance Electromagnetic Interference Shielding Material. Carbon.

[47] Wen C., Li X., Zhang R., Xu C., You W., Liu Z., Zhao B., Che R. (2022). High-Density Anisotropy Magnetism Enhanced Microwave Absorption Performance in Ti_3_C_2_T*_x_* MXene@Ni Microspheres. ACS Nano.

