# Supplementary material for: Bionic Aerogel with a Lotus Leaf-like Structure for Efficient Oil-Water Separation and Electromagnetic Interference Shielding
Source: Gels. 2023 Mar 10;9(3):214. doi: 10.3390/gels9030214 (PMC10048699; doi:10.3390/gels9030214)
Supplement: Supplementary file 1 [file gels-09-00214-s001.zip › gels-2259980-supplementary.pdf]

Bionic Aerogel with Lotus Leaf-like Structure for Efficient Oil-Water Separation and Electromagnetic Interference Shielding

Fengqi Liu, Yonggang Jiang *, Junzong Feng, Liangjun Li and Jian Feng *

Science and Technology on Advanced Ceramic Fibers and Composites Laboratory, College of Aerospace Science and Technology, National University of Defense Technology, Changsha 410073, China
* Correspondence: jygemail@nudt.edu.cn (Y.J.); fengj@nudt.edu.cn (J.F).

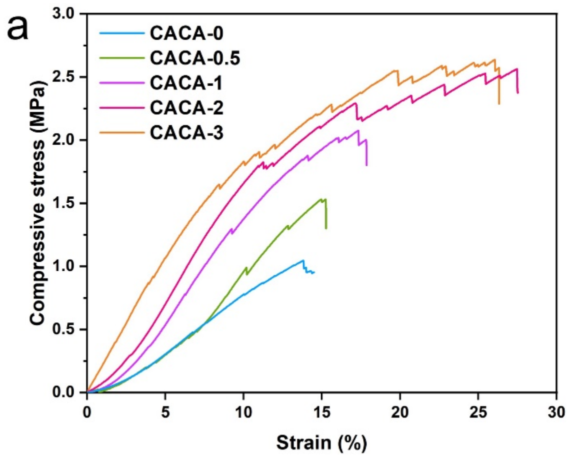

Figure S1. The compressive stress-strain curves of CACAs with different CNTs contents.

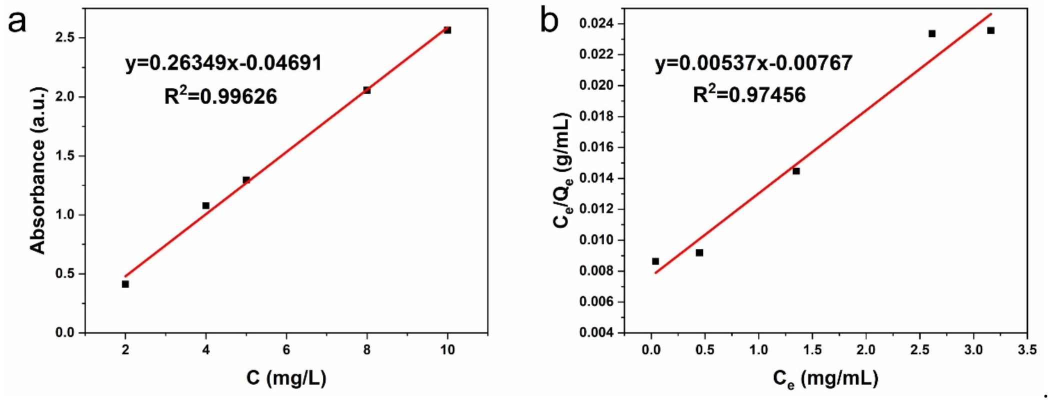

Figure S2. (**a**) Standard working curve of methylene blue. (**b**) Fitting curve of Langmuir adsorption isotherm equation.

Supplementary experimental section

1. Characterization

SEM micrographs were obtained using a field-emission scanning electron microscope (ZEISS Sigma 300, Germany). The high-resolution transmission electron microscopy (HRTEM) micrographs were recorded by a Tecnai G2 F20 (FEI, USA) microscope. Powder XRD patterns were recorded by a Empyren diffractometer (PANalytical, Netherlands) with Cu– Kα radiation (40 kV, 40 mA). Rietveld refinements of the measured XRD patterns in the 2θ range from 10 to 90° with a scanning rate of 0.2° min^{-1} were carried out using the general structure analysis (TOPAS) program (Bruker, Germany). Specific surface area and pore structure were measured with a surface area analyzer (3H-2000PM2, BeiShiDe Instrument, China) based on Brunauer-Emmett-Teller (BET) models. FTIR

spectroscopy was obtained with a Vertex 70 (Bruker) instrument. The compressive mechanical property was measured with a universal testing machine (FL4204GL, Fule Test Technology) with a displacement rate of 1 mm/min. The water contact angle and water infiltration processes were observed using a LSA100 (LAUDA Scientific, Germany) optical contact angle system. UV–vis absorption spectra were recorded with a UV-3600i Plus spectrometer (Shimadzu, Japan) at room temperature. The electrical conductivity of the film samples was measured by the four-probe method (2450, Keithley, USA). The EMI SE of the CANAs-CNTs composite (22.9 × 10.2 × 2 mm^3) was evaluated in the frequency range of 8.2–12.4 GHz (X-band) using a vector network analyzer (E5071C, Agilent) at room temperature by the coaxial method.

2. Measurement of methylene blue standard working curve

Methylene blue solutions at concentrations of 2, 4, 6, 8 and 10 mg/L were prepared. The absorbance of methylene blue solutions with different concentrations were measured at the maximum absorption wavelength (664 nm), and the linear regression equation was fitted to make the standard curve (**Figure S2a**).

3. Measurement of Langmuir adsorption isotherm curves

50 mL of methylene blue solutions with concentrations of 20, 40, 60, 80 and 100 mg/L were added into conical flasks containing 100 mg of CACAs, respectively. After stirring for 1 h, the absorbance of the solution was measured after removing CACAs by centrifugation. The Langmuir adsorption isotherm equation is defined as [41,42]:

$$C_e / Q_e = C_e / Q_m + 1/(K_L Q_m)$$

where Q_m is the maximum adsorption, C_e is the concentration of methylene blue at equilibrium, Q_e is the amount of methylene blue adsorbed in CACAs at equilibrium, and K_L is the equilibrium constant. The maximum adsorption amount of CACAs can be calculated according to the curve in **Figure S2b**.
